# Association between immune cells in peripheral blood and psychiatric symptoms

**DOI:** 10.3389/fpsyt.2023.1198734

**Published:** 2023-06-15

**Authors:** Juanjuan Yang, Qian Wang, Wei Jiang

**Affiliations:** ^1^Department of Health Management, The Second Affiliated Hospital of Xi’an Jiaotong University, Xi’an, China; ^2^Department of Oncology, The Second Affiliated Hospital of Xi’an Jiaotong University, Xi’an, China

**Keywords:** psychiatric symptoms, sleep, immunity, leukocyte, anxiety

## Abstract

**Background:**

There are bidirectional associations between immunological dysfunction and psychiatric symptoms. However, the associations between the levels of immune cells in the peripheral blood and psychiatric symptoms remain unclear. The present study aimed to evaluate levels of immune cells in peripheral blood in people with positive psychiatric symptoms.

**Methods:**

This retrospective study analyzed data from routine blood tests and psychopathology and sleep quality assessments. Data were compared between a group of 45 patients with *de novo* psychological symptoms and 225 matched controls.

**Results:**

Patients with psychiatric symptoms had higher white blood cell and neutrophil counts compared with controls. However, in a subgroup analysis, neutrophil counts were significantly higher than in controls only in patients with multiple psychiatric symptoms. Furthermore, monocyte counts were significantly higher in patients with multiple psychiatric symptoms than in controls. Further, sleep quality was lower in patients with psychiatric symptoms than in controls.

**Conclusion:**

White blood cell and neutrophil counts in the peripheral blood of patients with psychiatric symptoms were significantly higher and sleep quality was significantly lower than in controls. Participants with multiple psychiatric symptoms showed more significant differences in peripheral blood immune cell counts than other subgroups. These results provided evidence for the relationship between psychiatric symptoms, immunity, and sleep.

## 1. Introduction

Psychiatric disorders are an important public health issue and are considered to be important contributors to the total burden of disease; they are associated with suicide attempts, low quality of life, and social dysfunction (1–3). Recent studies have indicated that people who survived the first 30 days of infection with SARS-CoV-2, the virus responsible for the global COVID-19 pandemic, showed an increased risk of psychiatric disorders such as anxiety, depression, stress, and adjustment disorders (4). Therefore, it is important to prevent the onset of psychiatric disorders and develop new treatments to ameliorate the burden of psychiatric disorders.

Although the etiopathology of psychiatric disorders is complex and incompletely understood, more and more studies have suggested that dysfunction in immune and inflammatory responses may be involved in the onset and prognosis of psychiatric disorders (5, 6). A meta-analysis provided evidence that peripheral immune cells and inflammatory biomarkers were different in patients with psychiatric disorders, such as post-traumatic stress disorder, compared with control participants (7). Moreover, peripheral inflammatory cytokines can cross the blood–brain barrier and produce neuroinflammation, resulting in psychiatric disorders (8, 9). Finally, anti-inflammatory medications have been shown to reduce psychiatric symptoms (10). On one hand, immunological and inflammatory dysfunctions can promote the onset and prognosis of psychiatric disorders by altering the function of the neuroendocrine system (6). On the other hand, psychological factors, such as stress, can change the levels of immune cells and inflammatory cytokines in peripheral blood, also *via* the neuroendocrine system, and this can activate the sympathetic nervous system and hypothalamic–pituitary–adrenal axis (11–13). More experiments are needed to investigate the relationships between psychiatric disorders and the immune system.

The immune system is composed of immune cells and inflammatory factors and is the body’s main defense mechanism against pathogens. Immune cells and inflammatory factors circulating in the peripheral blood are important for maintaining the immune defense network (14). White blood cells (WBC) and subgroups of immune cells, such as neutrophils (NEUTA), lymphocytes (LYM), and monocytes (MONO), in peripheral blood are the major sources of inflammatory factors and provide a representation of the overall inflammatory status (15). Routine blood tests are efficient and inexpensive and can detect the overall inflammatory status; moreover, they do not require the use of expensive immunoassays (16).

Patients with psychiatric symptoms are more prone to have a low quality of sleep (17). Sleep disturbances, as stressors for the body, are associated with immunological dysfunction, which may be involved in the onset and prognosis of psychiatric symptoms (18). Psychological factors were shown to affect the number of immune cells in the peripheral blood (19, 20). Many studies have investigated changes in cytokine levels in the peripheral blood of patients with psychiatric disorders (5). However, the association between immune cell levels in the peripheral blood and psychiatric symptoms remains unclear. Moreover, the relationship between psychiatric symptoms, immunity, and sleep is also unclear. The present study aimed to evaluate potential changes in peripheral immune cell counts and sleep quality in people with positive psychiatric symptoms.

## 2. Materials and methods

### 2.1. Participants

All participants were recruited from the general population and underwent routine physical examinations. Data were collected from participants who had undergone this physical exam at the Second Affiliated Hospital of Xi’an Jiaotong University between 1 April 2021 and 15 June 2022. The inclusion criteria were: (1) psychological symptom tests and physical examinations performed in the Department of Health Management; (2) age ≥ 18 years; and (3) Han Chinese ethnicity. The exclusion criteria were as follows: (1) clinical metabolic conditions or chronic pathologies such as chronic obstructive pulmonary disorder, neurological conditions such as multiple sclerosis, and inflammation and infections such as with SARS-CoV-2; (2) the use of immunomodulatory drugs; (3) the use of psychiatric medications; (4) current pregnancy or breastfeeding; (5) the confirmed presence of malignant tumors; (6) C-reactive protein levels above 6 mg/L; and (7) a history of psychological symptoms. This retrospective study included 45 participants with psychiatric symptoms and 225 matched controls. This study was approved by the Ethics Committee of the Xi’an Jiaotong University College of Medicine and was conducted in accordance with the principles outlined in the Declaration of Helsinki.

### 2.2. Demographic and clinical data collection

Demographic and clinical data were extracted from electronic health records in the Department of Health Management and included age, sex, tobacco smoking history, systolic blood pressure (SBP), diastolic blood pressure (DBP), fasting blood sugar (FBS), body mass index (BMI), and medical history.

For blood tests, 3 ml of blood was extracted from every patient into ethylenediaminetetraacetic acid-coated tubes at room temperature. Routine blood tests were performed using an automatic blood cell analyzer (XN9000; Sysmex, Kobe, Japan) according to the manufacturer’s instructions. The data from routine blood tests, including WBC, NEUT, LYM, MONO, eosinophil granulocyte (EOS), and basophil granulocyte (BASO) counts were also collected. Participants in both groups were stratified by sex, age, presence of hypertension, diabetes diagnosis, and tobacco smoking history to compare differences in immune responses.

### 2.3. Assessment of psychopathology symptoms

The Symptom Checklist-90 (SCL-90) is a widely used tool for evaluating a broad range of psychopathology symptoms. It is a 90-item questionnaire designed to evaluate a broad range of psychopathology symptom dimensions including somatization, obsessive–compulsive, interpersonal sensitivity, depression, anxiety, anger hostility, phobic anxiety, paranoid ideation, and psychoticism (21). Each item is scored from 0 (*not at all*) to 4 (*extremely*) based on the severity according to the patient’s experience in recent weeks (22). The items relevant to each subscale symptom are averaged to obtain a subscale score, and all items are summed to obtain a total SCL-90 score. Higher scores reflect more severe symptoms; if the total score is more than 160, the number of positive items is more than 43, or any subscale psychopathology symptom score is higher than 2, patients are considered to have positive psychological symptoms (22). The Chinese version of the SCL-90 was validated and showed excellent psychometric properties (23, 24). In the present study, psychopathology symptoms were assessed using the Chinese version of the SCL-90.

### 2.4. Assessment of sleep quality

Sleep quality was assessed using the Chinese version of the Pittsburgh Sleep Quality Index (PSQI), a 19-item questionnaire designed to evaluate sleep quality (1). The total PSQI score ranges from 0 to 21, and higher PSQI scores reflect poorer sleep quality (1).

### 2.5. Statistical analysis

Statistical analysis was performed using SPSS software (v24.0; IBM Corp., Armonk, NY, United States). Continuous variables are presented as mean ± standard deviation, and categorical variables are presented as absolute values. The nonparametric Mann–Whitney U test was used to test differences between the continuous variables, and the chi-squared test was used to test differences in the categorical variables between the groups. The differences in immune cell counts and sleep quality scores among more than two groups were tested using the one-way analysis of variance followed by the Bonferroni *post hoc* test. Three models of linear regression were used to explore associations between SCL-90 scores and immune cell counts. Model 1 was not adjusted, model 2 was adjusted for sleep quality and model 3 was fully adjusted (age, sex, smoking, BMI, blood pressure, blood sugar, and sleep quality). Participants were divided into different subgroups on the basis of their SCL-90 subscale scores and the number of psychopathology symptoms. *p* < 0.05 were considered statistically significant.

## 3. Results

### 3.1. Demographic and clinical characteristics

A total of 45 participants experiencing *de novo* psychological symptoms (psychiatric group) and 225 matched controls (control group) were enrolled. The groups were matched by age, sex, history of tobacco smoking, SBP, DBP, FBS, BMI, and medical history. The demographic data and clinical characteristics of all participants are presented in Table 1. The mean age of the psychiatric group was 38.73 ± 14.39 years, and that of the control group was 41.04 ± 11.51 years; there was no difference between the groups (*p* = 0.185, Table 1). In the psychiatric group, 29/45 participants were women and 145/225 participants in the control group were women (Table 1). There was no statistical difference between the groups regarding hypertension (*p* = 1.000), SBP (*p* = 0.787), DBP (*p* = 0.113), diabetes (*p* = 0.722), FBS (*p* = 0.342), BMI (*p* = 0.289), or tobacco smoking history (*p* = 1.000; Table 1).

**Table 1:** Demographic and clinical characteristics of controls and psychiatric problems group.

Variable	Controls (*n* = 225)	Psychiatric problems group (*n* = 45)	*p*
Age (years)	41.04 ± 11.51	38.73 ± 14.39	0.19
Male (*n*/%)	80/35.6%	16/35.6%	1.00
Female (*n*/%)	145/64.4%	29/64.4%	1.00
Hypertension (*n*/%)	20/8.9%	4/8.9%	1.00
No Hypertension (*n*/%)	205/91.1%	41/91.1%	1.00
SBP (mmHg)	120.02 ± 14.80	120.31 ± 14.97	0.79
DBP (mmHg)	76.16 ± 10.68	73.33 ± 10.06	0.11
Diabetes (*n*/%)	12/5.3%	3/6.7%	0.72
No Diabetes (*n*/%)	213/94.7%	42/93.3%	0.72
FBS (mmol/L)	5.35 ± 1.61	5.14 ± 0.80	0.34
BMI (kg/m^2^)	23.53 ± 3.16	23.09 ± 3.46	0.29
Smokers (*n*/%)	25/11.1%	5/11.1%	1.00
No Smokers (*n*/%)	200/88.9%	40/88.9%	1.00

Continuous variables were presented as mean ± standard deviation (SD). BMI, body mass index; SBP, systolic blood pressure; DBP, diastolic blood pressure; FBS, fasting blood sugar.

Of the 45 participants in the psychiatric group, 22 had one positive psychological symptom, while the other 23 had more than one symptom. Of the identified psychiatric symptoms, 13 were somatization symptoms, 18 were obsessive–compulsive symptoms, 12 were interpersonal sensitivity symptoms, 23 were depression symptoms, 12 were anxiety symptoms, 16 were anger hostility symptoms, 8 were paranoid ideation symptoms, 7 were phobic anxiety symptoms, and 4 were psychoticism symptoms.

### 3.2. Association between immune cells in peripheral blood and psychopathology symptoms

We compared counts of immune cells in peripheral blood between the psychiatric and control groups. WBC counts were significantly higher in the psychiatric group than in the control group (*p* < 0.01; Table 2). As for subsets of WBCs in the peripheral blood, NEUT counts (*p* < 0.01) were significantly higher in the psychiatric group compared with the control group (Table 2). There were no differences in MONO, LYM, EOS, or BASO counts between the groups (Table 2).

**Table 2:** Comparison of immune cells in controls and psychiatric problems group.

Variable	WBC (10^9^/L)	NEUT (10^9^/L)	MONO (10^9^/L)	LYM (10^9^/L)	EOS (10^9^/L)	BASO (10^9^/L)
Controls	4.99 ± 1.00	2.78 ± 0.71	0.34 ± 0.09	1.73 ± 0.44	0.11 ± 0.13	0.03 ± 0.02
Psychiatric problems group	5.82 ± 1.55**	3.40 ± 1.33**	0.37 ± 0.10	1.90 ± 0.52	0.11 ± 0.11	0.03 ± 0.01
*p*	*P* < 0.01	*P* < 0.01	0.09	0.05	0.39	0.22

Continuous variables were presented as mean ± standard deviation (SD). WBC, white blood cell; NEUT, neutrophil; LYM, lymphocyte; MONO, monocyte; EOS, eosinophil granulocyte; BASO, basophilic granulocyte. *p < 0.05, **p < 0.01 versus controls.

**Figure 1:**
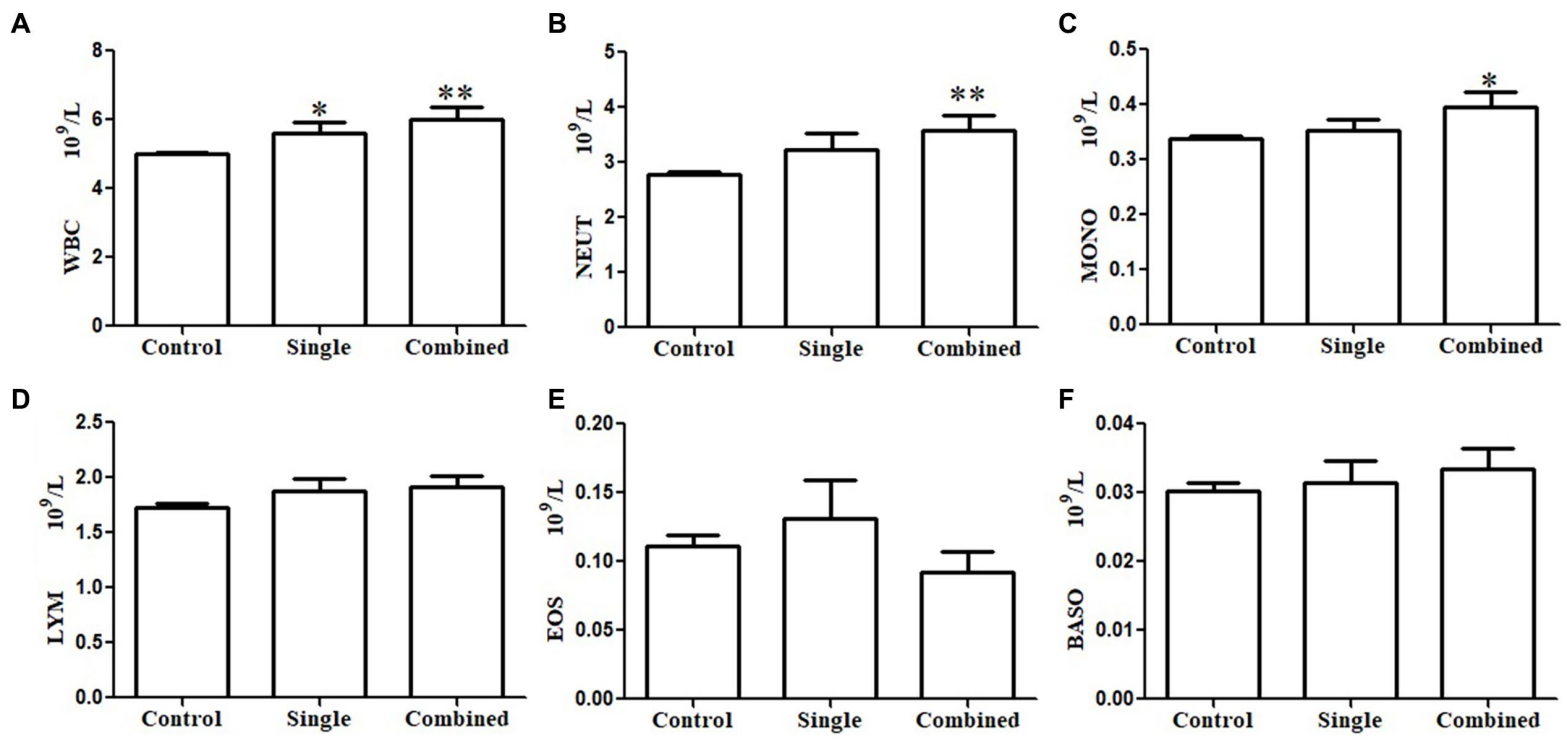
Subgroups analysis of the association between immune cells in peripheral blood and psychopathology symptoms based on the number of psychopathology symptoms. White blood cell (WBC) counts both in the single positive psychological symptom subgroup and combined multiple psychological symptoms subgroup were significantly higher than controls **(A)**. Neutrophil (NEUT) count **(B)** and monocyte (MONO) count **(C)** only in the combined multiple psychological symptoms subgroup were significantly higher than in the controls. There were no differences in lymphocyte (LYM) count **(D)**, eosinophil granulocyte (EOS) count **(E)**, and basophilic granulocyte (BASO) count **(F)** among the three groups.

As mentioned above, some participants showed a single psychological symptom while others showed multiple psychological symptoms. We investigated the association between immune cell counts in peripheral blood and psychopathology symptoms based on the number of psychopathology symptoms. Similarly, the WBC counts in the subgroup with a single psychological symptom (*p* < 0.05; Figure 1A) and the subgroup with multiple psychological symptoms (*p* < 0.01; Figure 1A) were significantly higher than in the control groups. However, NEUT counts were significantly higher (*p* < 0.01; Figure 1B) only in the subgroup with multiple psychological symptoms compared with the controls. Interestingly, MONO counts were also significantly higher (*p* < 0.05; Figure 1C) in the subgroup with multiple psychological symptoms compared with the controls. There were no differences in LYM (Figure 1D), EOS (Figure 1E), and BASO counts (Figure 1F) among the three groups. The results of the linear regression of the SCL-90 scores and immune cell counts with or without adjustment for sleep quality are presented in Table 3. The SCL-90 scores were positively associated with WBC counts in all models (all *p* < 0.01).

**Table 3:** Linear associations between SCL-90 score and levels of immune cells.

Immune cells	Model 1			Model 2			Model 3		
	Coefficient	SE	*p*	Coefficient	SE	*p*	Coefficient	SE	*p*
WBC	0.054	0.003	<0.01	0.012	0.004	<0.01	0.007	0.002	<0.01
NEUT	0.031	0.002	<0.01	0.008	0.002	<0.01	0.005	0.001	<0.01

WBC, white blood cell; NEUT, neutrophil; SE, standard error. Model 1 was not adjusted, model 2 was adjusted for sleep quality and model 3 was fully adjusted (age, sex, smoking, BMI, blood pressure, blood sugar and sleep quality).

**Figure 2:**
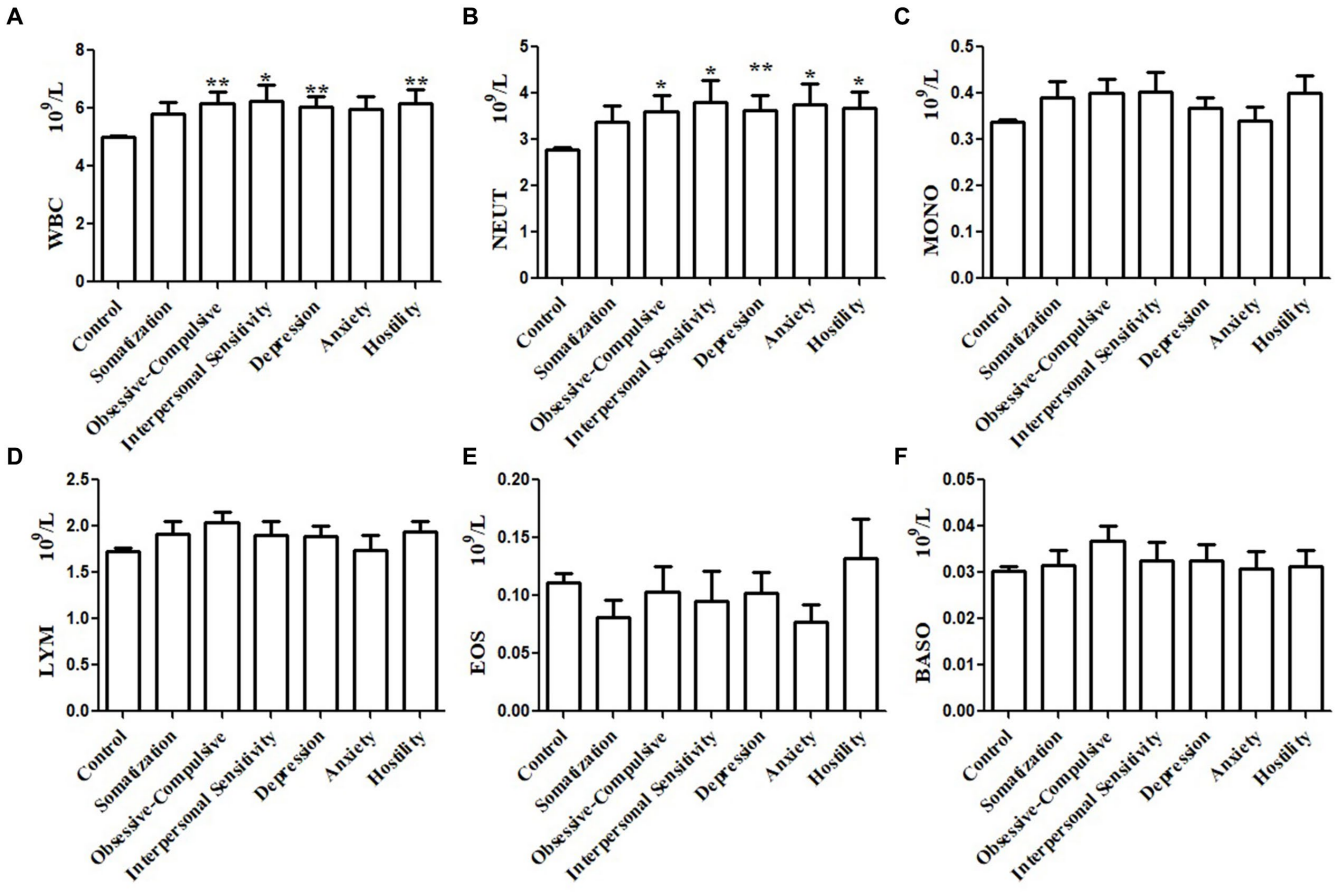
Subgroups analysis of the association between immune cells in peripheral blood and psychopathology symptoms based on subscale psychopathology symptoms. White blood cell (WBC) count in the peripheral blood of patients with obsessive–compulsive, interpersonal sensitivity, depression, or hostility was higher than that in the control group **(A)**. Neutrophil (NEUT) count in the peripheral blood of patients with obsessive–compulsive, interpersonal sensitivity, depression, anxiety, or hostility was higher than that in the control group **(B)**. The monocyte (MONO) count **(C)**, lymphocyte (LYM) count **(D)**, eosinophil granulocyte (EOS) count **(E)** and basophilic granulocyte (BASO) count **(F)** in participants with psychiatric symptoms were not higher than the controls.

We further investigated the relationships between immune cell counts in peripheral blood and certain psychopathology symptoms. Subgroup analyses were performed with subgroups of ten or more participants with a certain psychopathology symptom. Subgroup analyses were not performed for paranoid ideation symptoms, phobic anxiety symptoms, or psychoticism symptoms. Stratification analysis based on specific psychopathology symptoms showed that the WBC counts of participants with obsessive–compulsive, interpersonal sensitivity, depression, or hostility symptoms were significantly higher than the controls (Figure 2A). Similarly, the NEUT counts of participants with obsessive–compulsive, interpersonal sensitivity, depression, anxiety, and hostility symptoms were significantly higher than the controls (Figure 2B). The MONO (Figure 2C), LYM (Figure 2D), EOS (Figure 2E), and BASO counts (Figure 2F) did not differ between symptom subgroups and controls.

### 3.3. Association between sleep quality and psychopathology symptoms

**Figure 3:**
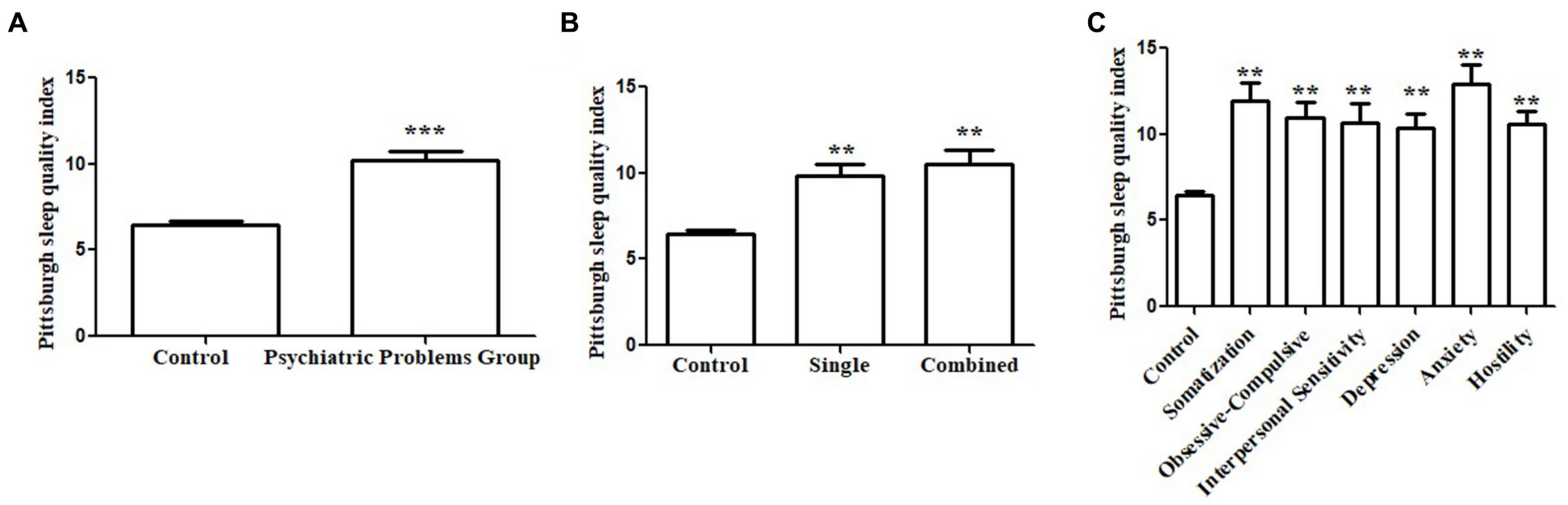
Association between sleep quality and psychopathology symptoms. Pittsburgh Sleep Quality Index (PSQI) score in the psychiatric group was significantly higher than the controls **(A)**. Subgroup analysis based on the number of psychopathology symptoms showed the PSQI scores were significantly higher than that of the control group **(B)**. Subgroup analysis based on the subscale psychopathology symptom showed the PSQI scores were significantly higher than that of the control group **(C)**.

The PSQI scores were significantly higher (indicating lower sleep quality) in the psychiatric group than in the control group (10.18 ± 3.68 vs. 6.46 ± 3.40, *p* < 0.001, Figure 3A). In stratification analysis based on the number of psychopathology symptoms (single/multiple; Figure 3B) or certain psychopathology symptoms (subgroups in 3.2; Figure 3C), the PSQI scores of each subgroup were significantly higher than those of the control group.

## 4. Discussion

The main objective of the current study was to examine associations between immune cell counts in peripheral blood and psychopathology symptoms. The most important finding of the present study was that the WBC and NEUT counts in participants with psychiatric symptoms were significantly higher than in control participants without psychiatric symptoms. Furthermore, the NEUT counts were significantly higher than the controls in the subgroup of participants with multiple psychological symptoms and not in the subgroup with a single symptom. Interestingly, the MONO counts were also significantly higher in the subgroup with multiple psychological symptoms compared with the controls. Similarly, stratification analysis of psychopathology symptoms showed that there were no differences in the MONO, LYM, EOS, or BASO counts between the psychiatric subgroups and the controls. This indicated that the changes in immune cell counts in participants with multiple psychiatric symptoms were more significant. Finally, the sleep quality of participants with psychiatric symptoms was significantly lower than that of the control participants.

Previous studies have reported that WBC counts and counts of WBC subsets increased in patients with psychiatric disorders. For example, a study by Lindqvist et al. reported that combat post-traumatic stress disorder was associated with elevated WBC counts (25). Euteneuer et al. reported that patients with major depressive disorder had higher NEUT and MONO counts (26). A previous study also reported that the NEUT–LYM ratio was higher in patients with bipolar disorder than in the control participants (27). Similarly, another study reported that the LEUK, NEUT, and MONO counts in patients with schizophrenia were significantly higher than in a control group (28). However, Garcia-Rizo et al. reported that patients with schizophrenia and the controls displayed similar mean values of WBC, NEUT, and MONO counts (29). Previous studies have all analyzed patients with psychiatric disorders. To our knowledge, the present study is the first to report an increase in WBC and NEUT counts in people with positive psychiatric symptoms.

The activation of the stress response has been consistently reported in psychotic disorders and has been reported to be associated with changes in immunological markers (15). Stress regulates the immune response through stress hormones such as epinephrine, noradrenaline, and corticosterone by activating the sympathetic nervous system and the hypothalamic–pituitary–adrenal axis (30). Heidt et al. reported that chronic variable stress increased the number of LEUK, NEUT, and MONO in circulation through the activation of upstream hematopoietic stem cells by releasing surplus noradrenaline, which signaled bone marrow niche cells to decrease CXCL12 levels through the beta3-adrenergic receptor (31). McKim et al. reported that social stress established a persistent extramedullary hematopoietic depot in the spleens of mice and produced multiple types of cells, including NEUT and MONO, by mobilizing hematopoietic stem progenitor cells from bone marrow engrafts into the spleen through beta-adrenergic signaling (32). However, stress changes the immune cells in peripheral blood by redistributing them among different body compartments through action from the sympathetic nervous system or hypothalamus–pituitary–adrenal cortex axis (11, 33).

Studies have suggested that immunological dysfunctions might be involved in the onset and prognosis of psychiatric disorders by altering neuroendocrine system function (6). First, stress was shown to promote the re-establishment of anxiety by trafficking MONO from peripheral regions to the brain after sympathetic activation (34). Second, peripheral inflammatory cytokines were shown to cross the blood–brain barrier and induce neuroinflammation, resulting in psychiatric disorders (8). Once peripheral immune cells and inflammatory cytokines are transmitted to the brain, these can activate the resident immune cells and induce neuroinflammation, which can lead to neuronal atrophy and synaptic pruning and alter the function of the neuroendocrine system (35). Finally, clinical trials have shown that anti-cytokine treatment had significant antidepressant effects when compared with control treatments (36). These results suggested that immunological dysfunction has the potential to induce psychopathology symptoms. Obtaining WBC counts is a cheap and readily available process and can be used as a reproducible biomarker of systemic inflammation that can be measured routinely. Thus, the WBC count may be useful as a potential marker of psychiatric disorders.

There are bidirectional associations between sleep quality and psychiatric symptoms. Early sleep problems may predict later psychiatric symptoms, and patients with psychiatric symptoms are more prone to have low sleep quality (17). Sleep disturbances, which are physical stressors, are associated with immunological dysfunction, which may be involved in the onset and prognosis of psychiatric disorders, as discussed above (18). Lower sleep quality in patients with psychiatric disorders may be due to smoking tobacco, drinking alcohol, or neuroendocrine dysfunction (1, 37). Sleep is important for regulating physical repair, immune function, and emotions (38). Poor sleep quality was associated with reduced immune function among college students (39). These results suggested that improving sleep quality may improve immune function and psychiatric symptoms. The present study found that immune cell counts in peripheral blood were higher and sleep quality was lower in participants with psychiatric symptoms when compared with control participants, supporting a relationship between emotion, immunity, and sleep.

The present study had several limitations. First, it did not address certain confounding factors, such as diet and life events. Second, the results provided evidence for a relationship between psychiatric symptoms, immunity, and sleep quality, but causality could not be inferred because of the cross-sectional study design. Moreover, the average immune cell counts were within normal ranges; therefore, these significant differences were all relative, and further studies are needed to investigate this clinically. Third, participants were selected according to SCL-90 scores/subscores and not definite diagnoses by a psychiatrist, which may reduce the specificity of the results. However, the heterogeneity in the participants was insignificant, and the results proposed an association between psychological symptoms and the immune system. Finally, subgroup analyses were not performed for paranoid ideation symptoms, phobic anxiety symptoms, or psychoticism symptoms because of a small sample size of participants positive for these symptoms. The present study revealed that certain immune cell counts in the peripheral blood of participants with psychiatric symptoms were higher than in controls, but the conclusions need to be verified in clinical studies.

## 5. Conclusion

The present study found that the WBC and NEUT counts in the peripheral blood of patients with psychiatric symptoms were significantly higher, while sleep quality was significantly lower, when compared with the control participants. These results provided evidence for a relationship between psychiatric symptoms, immunity, and sleep quality. WBC counts may be useful as a marker for psychiatric disorders, and improving sleep quality may improve immune function and psychiatric symptoms. More experimental studies are needed to explore these relationships. Further research is also needed to fully investigate the mechanisms that link psychiatric symptoms with the immune system and sleep quality.

## Data availability statement

The raw data supporting the conclusions of this article will be made available by the authors, without undue reservation.

## Ethics statement

This study was approved by the Ethics Committee of the Xi’an Jiaotong University College of Medicine and was conducted in accordance with the principles outlined in the Declaration of Helsinki. As for a retrospective study, written informed consent for participation was not required for this study in accordance with the national legislation and the institutional requirements.

## Author contributions

WJ conceived and designed the experiments. JY and QW collected the clinical data. JY, WJ, and QW analyzed the clinical data. JY and WJ wrote the paper. All authors contributed to the article and approved the submitted version.

## Funding

The present study was supported by National Natural Science Foundation of China (no: 82103022), Natural Science Foundation of Shaanxi Province (no: 2021JQ-410), and Science Foundation of the Second Affiliated Hospital of Xi’an Jiaotong University (no: RC(XM)202012).

## Conflict of interest

The authors declare that the research was conducted in the absence of any commercial or financial relationships that could be construed as a potential conflict of interest.

## Publisher’s note

All claims expressed in this article are solely those of the authors and do not necessarily represent those of their affiliated organizations, or those of the publisher, the editors and the reviewers. Any product that may be evaluated in this article, or claim that may be made by its manufacturer, is not guaranteed or endorsed by the publisher.
